# Analysis of an intergenerational service-learning experience based on physical exercise in a community setting: a mixed-method study

**DOI:** 10.3389/fpubh.2024.1509016

**Published:** 2025-01-14

**Authors:** Beatriz Alonso-Cortés Fradejas, Raquel Lafuente-Ureta, Sandra Calvo, Mario Fernández-Gorgojo, Jose Lesmes Poveda-López, Carolina Jiménez-Sánchez

**Affiliations:** ^1^Department of Nursing and Physiotherapy, Faculty of Health Sciences, University of León (Ponferrada Campus), Ponferrada, Spain; ^2^Department of Physiotherapy, Faculty of Health Sciences, Universidad San Jorge, Zaragoza, Spain; ^3^Aragón Health Research Institute, Zaragoza, Spain; ^4^Department of ysiatry and Nursing, Faculty of Health Sciences, University of Zaragoza, Zaragoza, Spain

**Keywords:** service-learning, intergenerational relations, exercise, aged, community participation

## Abstract

**Introduction:**

Physical activity offers numerous benefits that improve psychological well-being, reduce dependency, and foster intergenerational relationships. Universities play a key role in promoting the health of students by proposing actions that contribute to a sustainable future, fostering a mature society and reducing ageism. This service-learning project aimed to assess the impact of an intergenerational cane-walking program on older adults and physiotherapy students in a community setting. The project focused on promoting health and fostering intergenerational relationships.

**Methods:**

A concurrent, nested mixed-methods design was used for the intervention, involving intergenerational group walks during the 2022–2023 academic year. The program was designed and supervised by faculty members and three fourth-year fellows. Data collection was based on adherence to the intervention, the Behavioral Regulation in Exercise Questionnaire (BREQ-3), interviews with older adults and feedback questionnaires completed by them and participating students.

**Results:**

Satisfactory adherence was evidenced among older adults, with 65.79% of participants who completed the project and 72% who adhered to the intervention, although there were no statistically significant differences in terms of motivation to exercise taking that adherence into account. The project’s ability to foster intergenerational relationships was rated by the seniors at 9.50 ± 0.6, and 100% answered affirmatively about its capacity of contribute to improving their health and well-being. Student feedback also reflected high scores for fostering intergenerational relationships, with scores of 10 (fellows), 8.7 ± 1.2 (third-year students), and 8.27 ± 1.2 (second-year students). The project’s contribution to skills development was rated positively by 100% of the fellows, 88.6% of the second-year students, and 74.1% of the third-year students. In the nested study, three key themes related to exercise emerged by older adults: (1) perceptions of exercise, (2) barriers to exercise and (3) motivations for exercise. Regarding satisfaction with the program, three main themes emerged: (1) intergenerational relationships, (2) strengths of the program and (3) suggestions for improvement.

**Conclusion:**

The program appears to improve the well-being of older adults and provide valuable experiential learning for students. Thus, service-learning projects could effectively promote sustainable health practices, highlighting the important role of universities in community health initiatives.

## 1 Introduction

Demographic trends are changing worldwide, leading to an aging population. In 2050, the world’s population aged 60 and over will be 2.1 billion (1). In recent years, the concept of “Healthy Aging” has emerged as the optimal biological, sociological and physiological development throughout life (2). The Healthy Aging Strategy takes a life-course approach that aims to maximize the health and well-being of all older people (3).

Physical activity (PA) offers numerous benefits for aging at different levels. It has been proven that the combination of PA and spending time outdoors has a great impact on human health (4). Thus, in 2003, the concept of “green exercise” was born, which is any PA that takes place outside the home (4). When these activities are promoted in groups in the natural environment, functional and psychosocial benefits can also be achieved, fostering social relationships (5). Furthermore, some studies have concluded that motivation is an important aspect of practicing PA, with both instructors or healthcare professionals and positive reinforcement being essential (6). This is crucial to create a feeling of empowerment and enjoyment, in addition to the benefits of the PA itself, which also promotes adherence. The literature has shown that community-based group exercise programs for older people, especially in a natural environment, appear to promote both motivation and adherence over time (7). However, we must not overlook the barriers that are closely related to motivating older people, such as health problems, family responsibilities or education about the benefits and recommendations of PA, among others (8).

On the other hand, the mission of universities relates to the development of service to the community (9), in addition to the classic work of teaching and scientific research. Thus, universities must play a key role in promoting global health, proposing actions that contribute to a sustainable future, and promoting a mature society led by socially responsible professionals with ethical values and less ageism. Higher education institutions are also committed to meeting with the Sustainable Human Development (SHD), in which the paradigm of sustainability is a central concern for human existence, considering aspects such as development, cooperation, health, ecology, ethics, or global citizenship (10).

Higher education programs such as Service-Learning (SL) are being developed to build skills focused on community engagement at the university while supporting the achievement of several Sustainable Development Goals (SDGs) (10). In terms of methodological approach, a reciprocal relationship between service and learning is achieved, as SL combines service to the community with academic and experiential learning. In this way, SL enables students to approach their professional future through the development of social skills (11). Within SL, intergenerational SL involving students and older people is a valid option that enables health and social issues to be addressed. In these SL projects, in addition to the specific interventions of the service, a real interaction is created in which experiences from the daily lives of all participants are shared (12). In this way, a bidirectional befriending approach is created, which is particularly relevant to reduce ageism by students (13, 14).

Considering the need to support adherence to exercise among the older adult population and prepare future physiotherapists for the demands of an aging population, the aim of this SL project was to evaluate the impact of an intergenerational walking program developed in a community setting on a group of older adults (OAs) and students.

## 2 Materials and methods

### 2.1 Design

An experimental study was developed involving an intergenerational SL experience based on PA in a community setting.

A concurrent, nested, mixed-method design was selected for this study. It was conducted in accordance with the Declaration of Helsinki and following the guidelines of the Mixed Method Article Report Standards (MMARS) (15). The quantitative approach followed was a quasi-experimental design. Besides, a qualitative descriptive approach was explored using content analysis.

This study has been approved by the University Ethics Committee of León (ETICA-ULE-049-2022), ensuring participant privacy per Organic Law 3/2018 of December 5. The SL project is supported within the framework of the cooperation agreement signed between the Social Service of the Junta de Castilla y León, the University of León and the city councils of León and Ponferrada (Spain).

### 2.2 Participants and setting

The project included participants divided into two study groups, OAs and students, who signed the subsequent informed consent after agreeing to participate in the project, clearing all their doubts and accepting their participation in the focus group by recording their voice.

The initial sample of OAs, which consisted of 38 participants, was linked to the SL project thanks to an *Intergenerational Approach Program*, funded by the Social Services of the regional government, and developed in collaboration between the University and the local Council’s Department for Senior Citizens and Citizen Participation. This fact facilitated the project’s alignment with the SDG-17 (Partnerships for the Goals). Inclusion criteria for OAs were: (1) the ability to walk independently, (2) over 55 years of age, (3) no difficulty in performing moderate physical activities assessed with the Physical Activity Readiness Questionnaire (PAR-Q), (4) participation in practical walking training with canes and (5) the ability to read and complete questionnaires in Spanish language. Exclusion criteria were cardiovascular diseases that were incompatible with PA, which was determined by completing the PAR-Q.

The student sample included 71 participants: 27 third-year physiotherapy students as one of two possible activities within the continuous assessment of the subject “Specific methods in physiotherapy I” (Group A) and 44 s-year students as a compulsory activity within the face-to-face placements of the subject “Public health, health law and community physiotherapy” (Group B) at a Spanish university. Inclusion criteria were students enrolled in one of the two subjects associated with the SL project. In addition, 3 fourth-year fellows participated, one during the first and two during the second four-month period of the course, who were selected for the project following a selection process. Thus, a total of 74 students formed the posterior group of analysis.

### 2.3 Procedure

This SL project was integrated throughout the academic year 2022–23 into two subjects with learning competencies related to the field of health promotion within the physiotherapy degree program and took place over two consecutive semesters. The objectives, timetable and process of the project are described in Appendix.

With regard to the group of OAs, within the timeline, it is worth mentioning the participation in a specific three-hour training session on walking with canes by an instructor paid for the city council of Ponferrada (Spain). This happened after the research group had verified the absence of exclusion criteria and the informed consent, the protection of images and other questionnaires described below (sociodemographic data, chronic comorbidity information and Spanish Behavioral Regulation in Exercise Questionnaire-3) were completed.

On the students’ side, the SL project started in the first days of the two semesters with the recruitment of the fourth-year fellows and the holding of information sessions for all students involved on the main objectives we sought to promote: intergenerational relationships and development or reinforcement of competences, such as settlement in knowledge, and guidance to older participants, of the technical pillars of cane-walking and exercise plan with canes, respectful treatment and leadership capacity.

**Figure 1:**
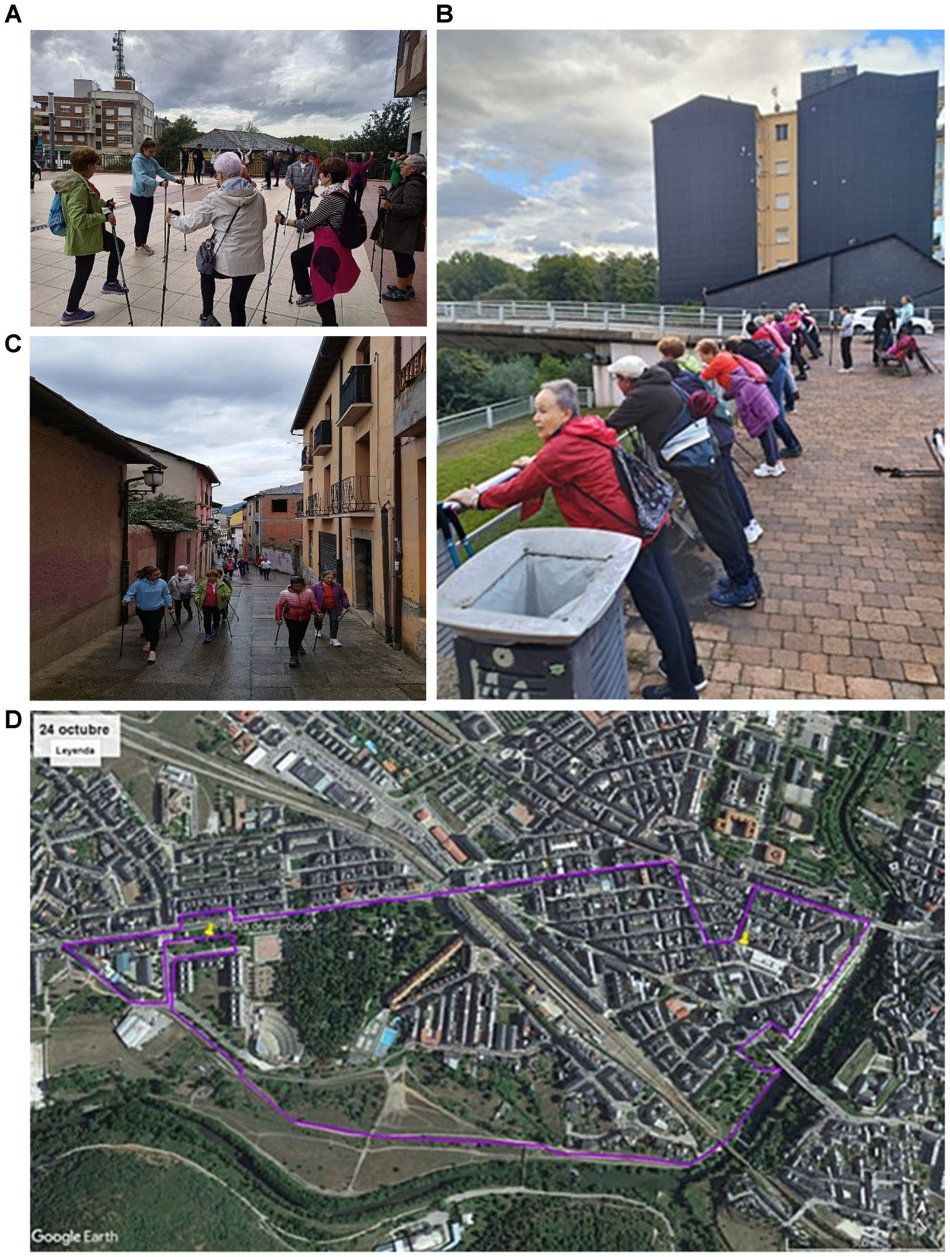
Intergenerational walking project photographs. **(A)** Intergenerational Walking Project 1 © 2022 by Beatriz Alonso-Cortés Fradejas is licensed under CC BY 4.0. To view a copy of this license, visit https://creativecommons.org/licenses/by/4.0/. **(B)** Intergenerational Walking Project 2 © 2022 by Beatriz Alonso-Cortés Fradejas is licensed under CC BY 4.0. To view a copy of this license, visit https://creativecommons.org/licenses/by/4.0/. **(C)** Intergenerational Walking Project 3 © 2022 by Beatriz Alonso-Cortés Fradejas is licensed under CC BY 4.0. To view a copy of this license, visit https://creativecommons.org/licenses/by/4.0/. **(D)** Intergenerational Walking Project 4 © 2022 by Beatriz Alonso-Cortés Fradejas is licensed under CC BY 4.0. To view a copy of this license, visit https://creativecommons.org/licenses/by/4.0/.

After reinforcement (Group A) or initiation (Group B) of training in walking with canes by the professor in charge, the students were divided into groups of 3 to 4 people and assigned to a single outing with OAs. During these outings, the students guided the seniors in performing a warm-up exercise (Figure 1A), followed by training in proper cane technique and a short strength training exercise in the middle of the route (Figure 1B). These exercises and the route (Figure 1C) were carefully planned by fourth-year students, who provided detailed information and explanatory pictures to the other students via email a week before the outing. The SL project was developed in the city of Ponferrada (Spain), which has 52,963 inhabitants, a sub-humid continental Mediterranean climate and an abundance of peri-urban green areas. The outings alternated between urban (Figure 1D) and peri-urban areas, which were carefully selected to provide novel and challenging environments to encourage engagement and participation. The intergenerational outing itself, along with the time spent on the dynamics of the SL project and learning walking technique with canes, was validated as a computable activity as part of a compulsory continuous assessment in Group A or with 5 h of a mandatory personal practicum (3 h for learning the technique of walking with canes and 2 h for the outing) in Group B.

Questions about the routes, the correct cane techniques, or the exercises were answered by the professor on the morning of the outing, during which practice canes were also distributed. The approximately two-hour walks were conducted from October 4, 2022, to May 22, 2023, on Monday afternoons (16:30–18:30). Before each session, the fourth-year fellows or professor checked the attendance list of the students and the OA and divided the seniors into small groups led by students. During the session, the teacher in charge assessed compliance (achieved or not achieved) with three basic aspects: appropriate guidance/instruction during the outing regarding technical aspects (such as cane walking pillars and exercise table), respectful interaction with older adult, and taking the leadership role.

Of the 27 outings initially planned, 22 were completed, but 5 had to be canceled due to inclement weather.

### 2.4 Outcome measurements

Sociodemographic data and chronic comorbidity information were collected at baseline as part of the initial screening process for OA participants (Table 1).

**Table 1:** General characteristics of older adults (*n* = 38).

General characteristics	Values
Age (mean ± SD)	72.00 ± 5.13
Gender [*n* (%)]	
Male	6 (15.79)
Female	32 (84.21)
Educational level [*n* (%)]	
No education. But I can read and write	7 (18.92)
Incomplete Primary (school)	3 (8.11)
First grade	1 (2.70)
Second grade/First cycle	10 (27.03)
Third grade/Second cycle	10 (27.03)
University	6 (16.22)
Civil status [*n* (%)]	
Single	2 (5.26)
Married	16 (42.11)
Widower	15 (39.47)
Divorced	5 (13.16)
Employment situation [*n* (%)]	
Active	2 (5.26)
Retired	36 (94.74)
Lives alone	20 (52.63)
Chronic disease	19 (50.00)
Type of chronic disease [*n* (%)]	
Arterial hypertension	7 (18.42)
Arthrosis	7 (18.42)
Diabetes	5 (13.16)
Others	6 (15.79)
Health perception [*n* (%)]	
Excellent	2 (5.26)
Very good	4 (10.53)
Good	27 (71.05)
Regular	5 (13.16)
Weekly physical exercise (mean ± SD) minutes	430.26 ± 213.42

SD, Standard deviation. General characteristics of older adults (*n* = 38) © 2023 by Beatriz Alonso-Cortés Fradejas is licensed under CC BY 4.0. To view a copy of this license, visit https://creativecommons.org/licenses/by/4.0/.

Throughout the project, adherence to the intervention was measured by two aspects postulated in the general exercise literature (16): the permanence in the project until its completion and the fidelity of the participants in the planned outings.

In addition, the motivation of OAs who completed the program was assessed using the Spanish Behavioral Regulation in Exercise Questionnaire-3 (BREQ-3) (17). This is an extended BREQ-2 questionnaire comprising 23 items with 6 subcategories to measure the behavioral regulatory styles: four for intrinsic regulation, based on questions about how they perceive PA in their daily life; four for integrated regulation, to find out if PA is part of their identity and if it is fundamental to them; three for identified regulation, where they evaluate the benefits and advantages of PA; four for introjected regulation, asking questions about guilt for not exercising, four for external regulation, where the respondent’s opinions influence the practice of PA; and four for amotivation, where motives for PA are not present (18–20). A five-point response scale was used to rate the items, ranging from 0 (not true for me) to 4 (very true to me). The score for each dimension was determined as the average value of items that constituted each subcategory.

The SL project’s ability to promote intergenerational relationships (0–10 Likert scales) was evaluated among the OAs who completed the project via an *ad hoc* opinion questionnaire, as well as a question on their perception (yes/no) of the project’s ability to improve their health and well-being. The last question sought to analyze the effectiveness of the intervention in terms of achieving SDG-3 (good health and well-being).

At the end of the study, the opinions of all student participants were also analyzed. An ad-hoc opinion questionnaire with a Likert scale from 0 to 10, was used to assess the effectiveness of the project in fostering intergenerational relationships and its usefulness in developing skills and abilities. These questions aimed to evaluate the impact of the intervention on the promotion of quality education, which is in line with the objectives of SDG-4 (Quality Education). In addition, an open-ended question was included to allow students the opportunity to provide highlights, suggestions for improvement or criticism of the project.

### 2.5 Nested qualitative study

For the qualitative study component, after the last walk with the students, the OAs were contacted and asked if they were interested in participating in a focus group. Three semi-structured focus group interviews were conducted on May 29 and 30, 2023 by R.L., C.J. and S.C., three experienced qualitative researchers. Each focus group interview consisted of 7–8 OAs and lasted one and a half hours, until reaching data saturation when participants added no new information to answer the questions. The semi-structured interviews were based on a predefined interview guide (the question guide is shown in Table 2).

**Table 2:** Guide of questions for the focal groups in older adults.

1. What type of physical exercise do you do in your daily life, taking into account the last week? 2. What motivates you to exercise? Do you consider exercising pleasurable/satisfying? Do you think that physical exercise is part of your daily life? Do you consider practicing physical exercise beneficial/important? At what level do you think it is beneficial? How do you feel when you do not do physical exercise/when you do not attend sessions? Do you think that the people around you influence you to exercise? How? What demotivates or limits you from exercising? 3. Have you been surprised by your physical ability? When students prepare routes, they assume that being older adults they will have a lower capacity and then the students are surprised. Do you think that your physical capacity has been undervalued and that you could have done more than what was proposed? Has the group helped you to improve and surpass the physical capacity that you started with? 4. Do you think your satisfaction with life/happiness is different since you have participated in the cane-walking program? in what sense, what has changed? 5. Opinion questionnaire What experiences and/or relationships have been maintained with the students during the sessions? What do you think about the participation of the students in the development of the project? Overall, do you think the project has helped foster intergenerational relationships? Do you think the program has been adapted to your needs? Would you change anything about the program?

Guide of questions for the focal groups in older adults © 2023 by Beatriz Alonso-Cortés Fradejas is licensed under CC BY 4.0. To view a copy of this license, visit https://creativecommons.org/licenses/by/4.0/.

### 2.6 Quantitative analysis

Due to the exploratory nature of the study, no sample size planning was done prior to the study.

Quantitative data analysis was carried out using the statistical package IBM SPSS for Windows, version 28 (IBM Corp, Armonk, NY, USA).

For the descriptive analysis, the mean and SD or the median and interquartile range and numbers (percentages) were used. The Shapiro–Wilk test was used to determine the normality of the quantitative variable. The Mann–Whitney U test was used for between-group comparisons. Statistical analysis was carried out at a confidence level of 95% and a statistical significance of *p* < 0.05 for all comparisons.

### 2.7 Qualitative analysis

Qualitative data was analyzed through content analysis. Audio-recorded focus group interviews were transcribed verbatim and pseudonymized. After that, transcriptions were reviewed by one researcher for accuracy. Two researchers independently read and coded the meaning units from the transcriptions using Atlas Ti 24. The codes were then grouped into categories, and these were condensed into themes. To ensure trustworthiness, codes, categories and themes were discussed reflectively between both researchers until reaching an agreement, a third independent researcher reviewed the themes and related narrations to confirm the analysis.

## 3 Results

### 3.1 Results of the quantitative study

#### 3.1.1 Quantitative results in older adults

The initial sample of OAs consisted of 38 participants, with the following characteristics (Table 1). The average age was 72 years, with a predominance of women of 84.21%; the distribution by educational level showed a higher frequency of 27.03% for second grade/first cycle and the same percentage for third grade/s cycle, followed by 18.92% with no education but with literacy, 16.22% for university and 2.70% for first grade. The participants were predominantly married (42.11%) or widowed (39.47%); 94.74% were retired, while 52.63% lived alone. Regarding the presence of chronic disease comorbidities, 50% of participants had at least one comorbidity; the most common comorbidities included hypertension (18.42%) osteoarthritis (18.42%) and diabetes (13.16%). When the participants were asked about their perception of their state of health, the results were 5.26% excellent, 10.53% very good, 71.05% good and 13.16% fair.

**Figure 2:**
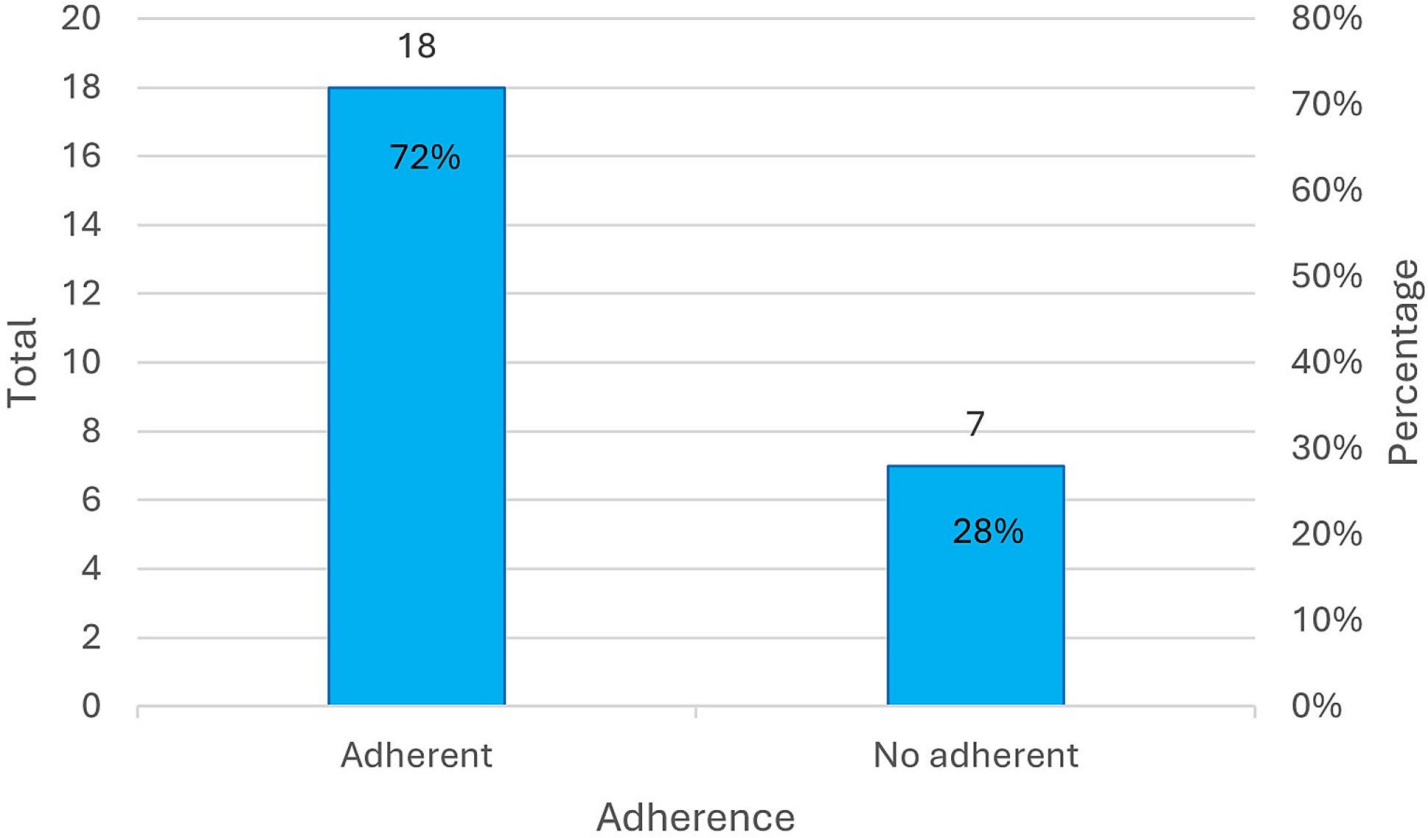
Distribution of the older adult participants according to adherence at the end of the intervention. Distribution of the older adult participants according to adherence at the end of the intervention © 2023 by Beatriz Alonso-Cortés Fradejas is licensed under CC BY 4.0. To view a copy of this license, visit https://creativecommons.org/licenses/by/4.0/.

The intervention demonstrated satisfactory adherence among the OAs, as indicated by the percentage of participants who completed the project—65.79% (25 out of 38)—and those who adhered to the intervention (Figure 2), defined as participants who completed at least two-thirds of the 22 outings (72%). On an individual level, the high adherence of three participants stands out, as they attended 100% of the sessions in the first semester, and two participants did so in the second semester.

The reasons given by participants who dropped out of the program (*n* = 13) were illness or serious injury (*n* = 6), prolonged absence from the city (*n* = 3), overlapping with other activities or family or personal problems (*n* = 3) or because they were unable to participate in the final assessment (*n* = 1).

The main reasons reported for absenteeism among the 25 participants who completed the intervention included caring for family members, attending medical appointments, and dealing with their own health issues.

When comparing the regulation of exercise behavior at the end of the intervention based on the adherence of the participants, no significant differences (*p* > 0.05) were found for the different BREQ-3 dimensions analyzed (Table 3). However, motivation scores for the final sample of OAs were more positive in the adherent group than in the non-adherent group, with intrinsic, integrated and identified regulation of PA close to the maximum score for all participants. In contrast, introjected regulation and external regulation scored low in both groups after the intervention. The amotivation dimension revealed favorable results in both groups.

**Table 3:** Motivation results according to the BREQ-3 questionnaire considering adherence after completion of the intervention.

BREQ-3 Subcategory	Adherent (*n* = 18)	Non adherent (*n* = 7)	*p*-value
Intrinsic regulation	3.67 ± 0.62 4 (0.31)	3.36 ± 0.67 3.5 (1.25)	0.357
Integrated regulation	3.38 ± 0.94 3.88 (1.06)	3.11 ± 0.96 3 (2)	0.534
Identified regulation	3.78 ± 0.41 4 (0.33)	3.57 ± 0.46 3.67 (1)	0.297
Introjected regulation	1.28 ± 1.10 1.38 (2.25)	1.36 ± 0.99 1.5 (1.5)	0.883
External regulation	0.38 ± 0.81 0 (0.75)	0.21 ± 0.30 0 (0.75)	0.883
Amotivation	0.44 ± 0.67 0(0.81)	0.54 ± 1.12 0 (0.75)	0.701
Mean ± SD Median (Interquartile range) Using Mann–Whitney *U* test			

Motivation results according to the BREQ-3 questionnaire considering adherence after completion of the intervention. © 2023 by Beatriz Alonso-Cortés Fradejas is licensed under CC BY 4.0. To view a copy of this license, visit https://creativecommons.org/licenses/by/4.0/.

General opinion of the SL projects was evaluated among the subjects who completed the intervention (*n* = 25) using an ad-hoc opinion questionnaire (Table 4), which showed scores of 9.50 ± 0.6 about ability to foster intergenerational relationships. 100% of the sample answered in the affirmative to the question of whether the project was able to contribute to improving their health and well-being (SDG 3).

**Table 4:** Students and older adults ad-hoc opinion questionnaire.

	Second-course students *n* = 44		Third-course students *n* = 27		Scholarship students *n* = 3		Older adults *n* = 25			
Do you think the project has helped to foster intergenerational relationships?										
0 Nothing	1	2	3	4	5	6	7	8	9	10 Very high
Mean ± SD	8.3 ± 1.5		8.7 ± 2.3		10		9.92 ± 0.40			
In general, the project has helped you to:										
1. Learn or fix some knowledge. 2. Develop, enhance or become aware of skills and abilities. 3. Develop, enhance or become aware of personal values.	Yes: 84.1% No: 15.9% Yes: 88.6% No: 11.4% Yes: 90.9% No: 6.8%		Yes: 81.5% No: 18.5% Yes: 74.1% No: 25.9% Yes: 88.9% No: 11.1%		Yes: 33.6% No: 76.3% Yes: 100% No: 0% Yes: 100% No: 0%		Improve health and wellbeing		Yes: 100% No: 0%	
Comment on what you liked the most and the least, as well as any ideas that we can include in future intergenerational projects to improve:										

#### 3.1.2 Quantitative results of the students

Table 4 also shows the results of the ad-hoc student opinion questionnaire, including a final open-ended response given to 74 participants. The data contained therein, as well as the quick compliance notes made by the professor in charge, show that the majority of the main objectives of the activity (appropriate guidance/instruction for the older adult, respectful interaction and leadership role) were met. Regarding the open-ended question, one third-year student noted in an open-ended response that his participation in the project “helped develop communication skills with future patients.” In addition, several second-year students emphasized that they found the “conversations with the seniors” and “the opportunity to learn from them” to be the most enjoyable aspects of the experience. However, some students suggested that a greater number of outings during the course could further enhance the program.

### 3.2 Results of the qualitative study

For the qualitative analysis 25 OAs (average age 72 years) participated in the focus group interviews.

The qualitative analysis of the *OAs in relation to PA*, revealed three main themes that complement and enrich the quantitative results: perceptions of PA, perceived barriers to regular participation in PA, and underlying motivational factors for PA. These themes not only support the questionnaire’s findings, but also provide a more nuanced understanding of the reasons behind the responses.

#### 3.2.1 Perceptions of PA

This theme emerged from the synthesis of OAs’ narratives related to their perceptions when performing PA.

The first category of this theme is titled “Type of PA they usually do,” and in it they explained the physical activities they usually did, most of them were very active and the most usual exercise for them was walking: *“Me, walk, walk, walk, walk. I also do gymnastics for a couple of days (…). Well, I did Taichi too, but this season my life got a little complicated and I have not been able to go.”* (P29, non-adherent).

The second category found in this theme is the OAs´ self-efficacy for PA. One of them explained that she was able to do more PA than they previously thought: *“Now I dare to do everything after not trusting myself in the first long runs and seeing that I did them perfectly. Now I dare with everything.”* (P18, adherent).

On the other hand, other participants explained their difficulties when doing PA: *“I have my difficulties walking, sometimes I cannot give more of myself because my leg does not allow me, so I cannot. Maybe at first, I run a lot and go among the first ones, but then I leave. Of course, because I cannot take it anymore. I have given so much of myself that I cannot take it anymore.”* (P34, adherent).

The last category of this theme synthetizes the narratives related to how the OAs felt when they did not exercise. They explain that they know how important PA is in their lives and that they feel bad when they do not exercise. *“If we want to grow old, we have to walk or do something. It’s just that if you do not exercise, you are missing something.”* (P17, adherent).

#### 3.2.2 Barriers to exercise

In this theme, the OAs expressed their perception of which elements prevented them from doing PA. This theme is composed of four categories.

The first category refers to health problems. The most common health problem was musculoskeletal pain: “*This year foot surgery has limited me.”* (P33, non-adherent).

The second category grouped the OAs´ narratives about how the COVID-19 pandemic restricted them to do exercise: *“Because I used to go to the gym, I went every day for 5 h, every day, before COVID pandemic.”* (P31, adherent).

Another common issue related as barriers to exercise was described in this third category: weather inclemency. The OAs explained that some weather conditions discouraged them to do PA: *“The activity of walk forces you to go out a little bit. Otherwise, you would not go out 1 day because it’s raining, another day because it’s cold, another day….”* (P10, non-adherent).

In a fourth category, the family burden is described: *“If I have to pay attention to something, I do exercise anyway because I try to do it beforehand. But if I have to take care of someone, then I do not go out exercising. For example, when I accompany my husband, it is the only thing that limits me.”* (P36, adherent).

#### 3.2.3 Motivations for exercise

The last theme that emerged deals with the motivations of OAs for exercise. This theme is composed of three categories.

The first category refers to the OAs´ well-being, considering physical, mental, and physical appearance dimensions: *“You get less tired. Yes, yes. You have different energy. Above all, the psychological thing (…) Not only the physical aspect of exercising but the psychological aspect (…) It clears your head.”* (P15, adherent).

The second category of this theme synthetizes the OAs´ feelings about their motivation in relation to other people, on the one hand, they highlighted the pleasure of socializing with people from the walking group: *“We are a family. Even though we do not know each other’s names.”* (P33, non-adherent).

Besides, they also explained the role of their relatives, who encouraged them to exercise: *“My children, they have encouraged me to walk. Yes, at first, I fell, and everything was so clumsy. And it was very good for me because I was also lazy and they told me come on, go out and go out. And I later became a widow, I also did not feel like going out. And if it wasn’t because of them, I would not go out and I’d be home all day. And my children come on, come out, it’s going to be well for you.”* (P25, adherent).

The last category, related to motivations for exercise concerns the routine of exercise in their daily life, in which the OAs explained how a planned PA in their daily life makes it easier for them to exercise: *“So, since I started, this is what you already have as a routine, you already say well, well now it’s over, but let us see, for next year you have to sign up.”* (P13, adherent).

The qualitative analysis of the *OAs general satisfaction* revealed three main themes that complement and enrich quantitative results: intergenerational relationships, strengths of the PA program and suggestions for improvement.

#### 3.2.4 Intergenerational relationships

This theme emerged as a result of the representation of the OA’s perceptions about the relationships that developed during the walking sessions with the young people.

The first category of this theme is related to breaking prejudices, where OA explained how young people had previous concerns about them: *“They said that they had been surprised by the physical condition we had (…). They thought we were going to be clumsier.”* (P18, adherent).

Besides, they also realized that they had a conceived idea of young people (“reverse ageism”): *“it often helps you change a little the idea you have about young people. Yes, because you have a preconceived idea and then you see them and say well, they are responsible. And they have concerns. Well, that contact makes you see it. Yeah.”* (P37, adherent).

Another category on this theme concerns the students’ perception of the support provided by the OAs. The OAs talked about how the students took care of them during PA sessions: *“They always accompany us, always, from the first in the group to the last. They always go with us, they do not leave anyone behind, they always go with us.”* (P17, adherent).

The last category of this theme deals with interpersonal relationships, in which the OAs reported on exchanges with young people: *“They seem interested in the things that I have done, for example, the Camino de Santiago and I have been explaining things about the route and they are very receptive and ask me and tell, oh, well, I want to do it. Very good.”* (P23, non-adherent).

#### 3.2.5 Strengths of PA program

This theme, which emerged during the analysis, represents the OAs´ thoughts on the benefits obtained during PA activity.

The first category of this theme related to the participants’ enjoyment during the program, with the OAs talking about the places they had discovered during the walk and the strength of the group: *“It is very well programmed. They are talking to you about all the things you see along the way. It becomes very enjoyable.”* (P15, adherent).

The second category or theme concerned participants´ satisfaction with the activity, with OA making the following comments: *“Well, you can say that it was a success. (…). Rating 10/10.”* (P34, adherent).

#### 3.2.6 Suggestions for improvement

On this theme, the OAs expressed their suggestions for improving the program for future editions. The first category related to organizational aspects, such as the location where the walks took place: *“The only thing I would ask for is to be more countryside than asphalt.”* (P27, adherent) or the best time to do the activity: *“Well, with the hour change perhaps the departure time in the afternoon, then it should also be delayed by an hour like the daytime, I think, especially in May.”* (P21, adherent).

The second category concerned the desire for an extension and continuity of the activity: *“We want it to continue.”* (P14, adherent).

The last category refers to the development of aspects that are more related to the methodology of walking with canes*: “I would like to advance in the theory of walking with canes. Someone should come 1 day and tell us to improve more or to level up.”* (P20, non-adherent).

## 4 Discussion

This study provides evidence of the impact of the intergenerational PA program in terms of adherence and satisfaction among OAs. In addition, positive motivation scores were found in relation to intrinsic, integrated and identified regulation, particularly among the participants who adhered to the program. For students, the program played a critical role in developing essential skills, and both OAs and students emphasized its effectiveness in fostering intergenerational relationships. The qualitative data further revealed an increased sense of empowerment through PA, highlighting the significance of these findings. This underscores the program’s broader benefits, not only in promoting physical well-being but also in enhancing emotional and social connections across generations.

To our knowledge, the present study is the first study to explore the effects of a PA intervention including cane walking conducted in OAs and students in the context of a SL project using a nested, mixed-method design.

Furthermore, it is worth mentioning that the intervention has achieved effective alignment with the proposed SDGs, in particular Goal 3 (Good Health and Well-being), Goal 4 (Quality Education) and Goal 17 (Partnerships for the Goals).

Satisfactory compliance with the intervention was noted for the OAs, which was reflected in the high completion rates and continued participation of those who remained engaged. This approach to measuring adherence, considering both the percentage of participants who completed the project and the attendance rate of participants, aligns with the framework proposed by Martin and Sinden (21). They suggest that adherence should be assessed by the number of sessions attended and the proportion of participants who completed the program, taking into account the duration and intensity. Furthermore, Hawley-Hague et al. (22) emphasize that compliance should not only be measured by the number of dropouts, but rather by a comprehensive evaluation of participant’s engagement throughout the intervention.

It is also worth noting that this higher adherence was achieved despite the considerable total duration of the project (27 weeks), which, according to Vseteckova et al. (23), is a presumed handicap because poorer adherence results were achieved in a long-duration exercise programs compared to a short-duration. The results of the present study, which showed a dropout rate of 34.21%, are not consistent with the data of Sluijs et al. (24), who found that long-term exercise programs can reach a level of 70% poor compliance.

The remarkable adherence reached in both at the level of the individual periods counted and at the total level, is of particular interest because it has been achieved in a population with a special idiosyncratic population (mainly older adult women), given their frequent dedication to tasks such as caring for others and medical surveillance of their own health. Engberg et al. (25) found that OAs perform less PA than would be desirable due to the simultaneity of events in their lives. However, evidence suggests that enjoyment plays a critical role in promoting adherence to PA programs (26). In this context, the high adherence observed in our study may be attributed to the participants’ enjoyment of intergenerational interactions. Notable behaviors such as smiling, laughing, maintaining eye contact, and participating in dynamic conversations with younger individuals likely contributed to the social and emotional appeal of the program, thereby fostering sustained engagement (27).

It is also possible that greater PA enjoyment influenced individuals’ self-reported ability to engage in regular PA (28). This increase in self-efficacy observed in the qualitative analysis of OAs may have helped to reduce barriers to PA and promote adherence. Self-efficacy is closely related to overcoming barriers and is an important predictor of PA participation (29–31). Our results support this relationship and show that OAs were able to engage in more PA than they had previously thought possible.

In fact, it is important to note that in this study, the participants in the group that adhered to the program showed more positive motivation scores than those that did not adhere to the program. This suggests that the participants who remained engaged were generally more motivated. However, as the qualitative findings indicate, absenteeism from some sessions in both groups was justified by unforeseen factors such as attending medical appointments, which led to absenteeism and subsequently reduced adherence in some participants, or other external barriers such as family burden, physical health problems, unfavorable weather conditions or even the ongoing effects of COVID-19, which were possibly the main barriers to their further participation, more than the motivation itself. These findings are consistent with Lees et al. (32), who also identified lack of time and health-related problems as two of the most significant barriers to participation in PA among the OAs included in its study. This is also in line with what is postulated by Camp et al. (33), who reported that the dependence on health factors increases with age and that the ability to adhere to exercise is influenced by several psychological constructs.

In agreement with Gladwell et al. (4) and Hanson and Jones (5), performing cane walking in natural outdoor environments may have provided some of the best health benefits for participants by improving their PA levels, restoring mental fatigue and improving mood and self-esteem. These findings are supported by the qualitative results about exercise motivation. In addition, “the group” promotes the social aspect that some people crave, and which may also have facilitated the observed adherence. Regarding outdoor and indoor exercise, a systematic review found no greater benefit of outdoor exercise compared to indoor exercise, although there was evidence that participants reported greater active valence and enjoyment of exercise (34). On the other hand, a recent review concluded that there is low-certain evidence that home-based exercise programs could provide benefits for OAs (35). Future studies comparing intergenerational indoor and outdoor exercise programs through SL would be needed.

Regarding motivation for the PA, positive motivation scores were found in relation to intrinsic, integrated and identified regulation particularly for the adherent group. This is in line with the study by Jofré-Saldía et al. (36) in which a multicomponent progressive training program for OAs was compared with no training program, showing improvements in the dimensions of the intrinsic and identified regulation BREQ-3 in favor of the training group. Furthermore, these findings aligned with the work of Picorelli et al. (37), who emphasize the importance of positive environmental factors and support systems in fostering motivation. Additionally, Cohen-Mansfield (38) highlight the motivational benefits of structured intergenerational programs, which enhance compliance and promote long-term participation. This intrinsic regulation is largely driven by the pleasure that the individuals derive from learning, with this enjoyment serving as the primary motivator for their actions (39). This is also supported by the qualitative data in which OAs suggested improvements to the program, reflecting a desire for progress that acts as a powerful motivator for continued participation.

Our findings highlight the complex interplay of motivational factors that influence participation in intergenerational PA programs among both older and younger participants. In the Cohen-Mansfield (38) study on - Motivation to participate in intergenerational programs - it was reported that older adults often reported motivations related to life circumstances, such as seeking support, companionship, or filling available time, whereas younger participants were driven by altruism, a sense of obligation, or perceived occupational value. Of particular note is that common motivations such as intergenerational interaction, enjoyment and friendship developed into enduring drivers over the course of the programs. This is consistent with the concept of identified regulation, which received higher scores in our study. Participants began to internalize the importance of the activity and embed it into their personal goals and values. This motivates them to engage in the activity, even if it is not inherently enjoyable (40). By identifying with the activity, individuals consciously integrate it into their value system, thereby enhancing their perceived autonomy (41, 42). Furthermore, the dynamic nature of the observed— motivations - in which intrinsic motivators such as satisfaction increased in importance over time — underscores the importance of a program design that fosters fun and meaningful connections. Future research should examine how these shifts in motivation influence long-term outcomes while identifying strategies to maintain engagement in different settings.

Quantitatively, this study found that the OAs in the adherent group scored slightly better than those in the non-adherent group in terms of identified regulation and slightly lower in terms of amotivation. These results are in line with those of Teixeira et al. (43), who suggest that integrated regulation is crucial for maintaining behavior over time, particularly when obstacles interfere with behavioral regulation or when activities become more difficult. A person who is motivated in an integrated way is more likely to exercise in the rain than a person who only exercises for pleasure (intrinsic reasons). On the other hand, high amotivation scores are associated with less exercise and reduced self-control, suggesting potential non-compliance with structured exercise interventions, as was the case in our study, in which the group that did not adhere to the program scored slightly higher on amotivation (33). These findings are supported by the perceptions and motivations for exercise reported qualitatively by OAs who have made PA a commitment and routine due to its multiple benefits. They also indicated that they are often encouraged to exercise by their children or other family members, which reinforces their commitment to PA.

Regarding SL projects, one of the main results found in this study, was the usefulness of an intergenerational experience based on SL methodology and the practice of a healthy habit to foster relationships between OAs and students of different levels. This is also reflected in the qualitative data that also demonstrates this strong relationship. The high score in promoting intergenerational relationships and the general satisfaction of both OAs and students suggest that the program has successfully created a supportive and enjoyable environment. The qualitative data revealed that satisfaction resulted, not only from the PA program, but also from discovering new people or places during the walks, having a scheduled PA in their daily life which facilitates them to exercise, and the power of the group, all of which contributed to the positive experiences. These findings are consistent with the Cohen-Mansfield study, which emphasized that motivation is often driven by positive intergenerational relationships and enjoyment. They concluded that activities that focus on a broader range of topics could encourage greater participation among older adults. And that incorporating intergenerational programs into diverse settings and promoting these programs as opportunities to support and interact with others could be an effective motivator for younger participants (38). Other studies have also found effects of a SL program among students and OAs, highlighting the intergenerational component as an essential element in reducing the gap between generations (12, 44).

In terms of the impact of the intervention on intergenerational relationships, some OAs in the focus groups emphasized that the program had helped them recognize the initial underestimation of students’ ability to exercise. This is consistent with previous literature that has documented evidence of ageism against older exercisers (45), which is also highlighted in the Global Report on Ageism (46). To address this public health issue, the report recommends intergenerational interventions, such as those undertaken in this study, as a key strategy alongside policy changes and educational initiatives. However, this project found not only discrimination against OAs, but also the so-called “reverse ageism,” which according to Raymer et al. (47) is due to the fact that the hype about generational differences gives credence to reverse-ageist ideologies, and that such ideologies engender reverse-ageist discriminatory behaviors directed at young professionals.

The positive feedback from students regarding the project’s ability to foster intergenerational relationships and develop valuable skills underscores the importance of SL programs in higher education. These findings are supported by Dauenhauer et al. (48) and Canedo-García et al. (49), who emphasize the educational and social benefits of intergenerational activities.

Another significant achievement of our research is the intervention’s success in enhancing the skills and abilities, demonstrating the fulfillment of SDG-4 (Quality Education). This finding aligns with Dauenhauer, Steitz, Aponte, and Faria (2010) (48), who argued that relationships established with OA in SL through PA, have a strong educational component. Furthermore, studies on intergenerational SL conclude that students who participate in such programs not only feel more competent in interacting with OAs, but also develop skills that are important for their future careers (14, 50).

Other studies concluded that students who develop SL considered themselves more competent in their interactions with OA after interacting with them (14) and even develop their future professional skills (50). Building intergenerational relationships has the potential to promote healing and growth and enable engagement for mutual benefit.

The main limitations of our study are the small sample size and the convenience random selection. Although authors such as Jansons et al. (51) and Allen et al. (52) argue that voluntary participation in studies, as was the case in our study, favors adherence, this may have led to biases in the proportions and main themes identified in the focus groups. Besides, the questions asked in the qualitative design are closely related to the quantitative data collection, this could bias the perspectives of the OAs.

We believe that future research on intergenerational PA practices should further analyze into the capacity of such interventions to reduce ageism among all the groups involved, as well as negative attitudes toward aging and reverse ageism.

## 5 Conclusion

The intergenerational walking program showed remarkable benefits for both OAs and students. The OAs’ adherence to the program was featured despite the handicap of its long duration. The OAs reported high satisfaction, highlighting the program’s effectiveness in promoting physical activity and well-being. It was also found that the motivation scores for intrinsic, integrated and identified regulation were more positive in the adherent group than in the non-adherent group. Students also expressed positive feedback regarding the development of skills and intergenerational relationships, supporting the value of SL programs in higher education.

Future programs should continue to leverage the benefits of intergenerational activities and consider the motivations and barriers identified in this study to foster supportive environments and healthier and more engaged communities. Furthermore, the integration of SL into higher education curricula represents a challenge for the future, as it ensures the acquisition of professional and experiential skills for students and promotes the development of social sustainability within the community.

## Data Availability

The raw data supporting the conclusions of this article will be made available by the authors, without undue reservation.
