# Supplementary material for: Analysis of an intergenerational service-learning experience based on physical exercise in a community setting: a mixed-method study
Source: Front Public Health. 2025 Jan 14;12:1509016. doi: 10.3389/fpubh.2024.1509016 (PMC11803859; doi:10.3389/fpubh.2024.1509016)
Supplement: Supplementary file 1 [file Table_1.pdf]

Appendix. Objectives, timetable and process of the project.

<table>
<tr><td rowspan="2">Aims of the project</td><td>Students</td><td>Older adults</td></tr>
<tr><td>- Promote intergenerational relationships.

- Contribute to the development or strengthening of the following skills:

1. Provide knowledge and guidance to older participants on the technical fundamentals of pole walking and the exercise plan with poles.

2. Respectful interaction.

3. Leadership skills.</td><td>- Promote intergenerational relationships.

- Promote the perception of improved health and well-being.

- Promote adherence to the group practice of walking with canes over an academic year.</td></tr>
<tr><td colspan="2">Timeline</td><td>Process</td></tr>
<tr><td colspan="2">Second four-month period 2021-22</td><td>Walking training with canes (3 hours personal credit) - second-year physiotherapy students (including future Group A students).</td></tr>
<tr><td colspan="2">First week of September 2022</td><td>Application by the Ethics Committee.

Recruitment for participation of third-year physiotherapy students and fourth-year physiotherapy fellows.</td></tr>
<tr><td colspan="2">Second week of September 2022</td><td>Explanatory meeting with older adults.

Handing out an information sheet.

Filling out the PAR-Q.</td></tr>
<tr><td colspan="2">Third week of September 2022</td><td>Signed informed consent forms from older adults (n=38), Group A students and fourth-year physiotherapy fellows.

Completion of initial questionnaires for older adults.</td></tr>
<tr><td colspan="2">Last week of September 2022</td><td>Walking training with canes - older adults and fourth-year physiotherapy fellow.</td></tr>
<tr><td colspan="2">4&10 October 2022</td><td>Outings with older adults, fourth-year physiotherapy fellow and professor.</td></tr>
</table>

		Walking with poles training reminders- Group A students (third year).
**17 October-19 December 2022**		Outings with Group A students, older adults, fourth-year physiotherapy fellow and professor.
Exams period	9 January-6 February 2023	Completing opinion questionnaires - Group A students.<br><br>Outings with older adults, fourth-year physiotherapy fellow and professor.
13&20 February 2023		Rest period outings with older adults.<br><br>Recruiting new fourth-year physiotherapy fellows to participate.<br><br>Walking training with canes and signed consent forms - - new fourth-year physiotherapy fellows.
27 February-3 March 2023		Outings with older adults, fourth-year physiotherapy fellows and professor.<br><br>Walking training with canes - Group B students.<br><br>Signed consent forms - Group B students (3 hours personal credit).
**6 March 2023-22 May 2023**		Outings with Group B students (2 hours personal credit) and older adults.
29-30 May 2023		Completing opinion questionnaires - Group B students.<br><br>Completing final questionnaires - older adults.<br><br>Conducting focus groups with older adults
